# Lung Persistence, Biodegradation, and Elimination of Graphene‐Based Materials are Predominantly Size‐Dependent and Mediated by Alveolar Phagocytes

**DOI:** 10.1002/smll.202301201

**Published:** 2023-06-01

**Authors:** Thomas Loret, Luis Augusto Visani de Luna, Matteo Andrea Lucherelli, Alexander Fordham, Neus Lozano, Alberto Bianco, Kostas Kostarelos, Cyrill Bussy

**Affiliations:** ^1^ Nanomedicine Lab 2.0 School of Biological Sciences Faculty of Biology Medicine and Health The University of Manchester Manchester Academic Health Science Centre Manchester M13 9PT UK; ^2^ National Graphene Institute The University of Manchester Manchester M13 9PL UK; ^3^ Lydia Becker Institute of Immunology and Inflammation Faculty of Biology Medicine and Health The University of Manchester Manchester Academic Health Science Centre Manchester M13 9PT UK; ^4^ CNRS Immunology Immunopathology and Therapeutic Chemistry UPR 3572 University of Strasbourg ISIS Strasbourg 67000 France; ^5^ Catalan Institute of Nanoscience and Nanotechnology (ICN2) CSIC and BIST Campus UAB Bellaterra Barcelona 08193 Spain

**Keywords:** alveolar phagocytes, carbon materials, clearance, degradation, in vivo, toxicity

## Abstract

Graphene‐based materials (GBMs) have promising applications in various sectors, including pulmonary nanomedicine. Nevertheless, the influence of GBM physicochemical characteristics on their fate and impact in lung has not been thoroughly addressed. To fill this gap, the biological response, distribution, and bio‐persistence of four different GBMs in mouse lungs up to 28 days after single oropharyngeal aspiration are investigated. None of the GBMs, varying in size (large versus small) and carbon to oxygen ratio as well as thickness (few‐layers graphene (FLG) versus thin graphene oxide (GO)), induce a strong pulmonary immune response. However, recruited neutrophils internalize nanosheets better and degrade GBMs faster than macrophages, revealing their crucial role in the elimination of small GBMs. In contrast, large GO sheets induce more damages due to a hindered degradation and long‐term persistence in macrophages. Overall, small dimensions appear to be a leading feature in the design of safe GBM pulmonary nanovectors due to an enhanced degradation in phagocytes and a faster clearance from the lungs for small GBMs. Thickness also plays an important role, since decreased material loading in alveolar phagocytes and faster elimination are found for FLGs compared to thinner GOs. These results are important for designing safer‐by‐design GBMs for biomedical application.

## Graphical abstract

This work highlights the importance of dimensions in the design of safe graphene‐based nanomaterials. Decreased thickness leads to increased loading in alveolar macrophages, resulting in slower elimination. Large sheets are internalized with difficulty and are poorly degraded in alveolar phagocytes in comparison to small sheets, resulting in increased tissue damages and long‐term persistence in lungs.

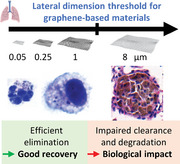

## 1 Introduction

The respiratory system is a major portal of entry for microorganisms and xenobiotics. Exposure to airborne pollutants and microorganisms can cause chronic lung inflammation and diseases such as asthma, fibrosis, or cancer and lead to impairment of lung functions or even death.^[^ ^1^, ^2^ ^]^ Targeting the respiratory system by direct administration of pharmaceuticals into the lungs is therefore representing a highly valuable approach to improve health outcomes. However, controlling the deposition of inhalable drugs in the respiratory tract is quite challenging. In this context, drug delivery systems based on nanomaterials offer new opportunities for enhanced delivery and efficacy.^[^ ^3^, ^4^ ^]^ Such improvement has been demonstrated recently with a liposomal formulation of antibiotics that has been commercialized to target pulmonary macrophages infected by mycobacterium.^[^ ^5^ ^]^

Although most drug delivery systems are currently based on liposomal formulation,^[^ ^6^ ^]^ two‐dimensional (2D) materials such as graphene‐based materials (GBMs) have recently emerged as new drug delivery candidates owing to their unique combination of physicochemical and biological properties. On the one hand, their high surface area to volume ratio is a major feature allowing not only high binding capacity,^[^ ^7^ ^]^ but also the grafting of various moieties on their surface for multimodal/multifunctional capability.^[^ ^8^ ^]^ On the other hand, GBMs have the capacity to enter in various cell types and ability to biodegrade, two essential features for a successful intracellular delivery.^[^ ^9^, ^10^ ^]^ Among the most common types of GBMs explored for biomedical application is graphene oxide (GO). GO is an amphiphilic material that possesses a surface rich in oxygen‐containing groups conferring stability in aqueous suspension.^[^ ^7^, ^11^ ^]^ Due to its amphiphilic nature, binding of both hydrophilic and hydrophobic molecules to GO via covalent and non‐covalent functionalization is possible. In comparison, graphene, whether it is categorized according to the number of layers as graphene nanoplatelets (GNPs) or few‐layer graphene sheets (FLGs), is hydrophobic, as it possesses only sp^2^ carbon regions. For this reason, it needs to be chemically functionalized or exfoliated to become stable in aqueous suspension. Although chemical functionalization of either GO or FLG can improve specificity and bioavailability, it may compromise the biocompatibility of such drug delivery systems.^[^ ^7^ ^]^ Prior to the translation of drug delivery systems based on GBMs into clinical practice, a comprehensive evaluation of their safety profile would therefore be critical to address all health concerns and fulfil regulatory requirements, especially when considering pulmonary application.^[^ ^12^, ^13^ ^]^

In respect to inhalation, the pulmonary impact of GBMs have been assessed before,^[^ ^14^ ^]^ albeit primarily for the purpose of replicating unintended exposure to airborne GBMs at the workplace. In several in vivo studies, single pharyngeal aspiration of GNPs or FLGs was demonstrated to induce acute lung inflammation followed by progressive tissue recovery by day 7 or 28.^[^ ^15^, ^16^, ^17^ ^]^ Importantly, regardless of the GBMs used in these studies, there was no tissue remodeling, although most materials were shown to persist in the respiratory tract. Conversely, Schinwald et al. highlighted that large and thick GNPs may be responsible for frustrated phagocytosis in lung macrophages, a predictive indicator often leading to tissue remodeling and fibrotic lesions, but found no evidence of long term damages despite the material biopersistence in a follow‐up study.^[^ ^15^, ^18^ ^]^ In another study, Park et al., reported biological effects in lungs for up to 90 days after administration of GNPs, including an increase in the number of recruited cells, elevated levels of pro‐inflammatory mediators, and an increase in apoptotic cells in broncho‐alveolar lavage (BAL) fluids.^[^ ^19^ ^]^ In contrast, no significant inflammation was surprisingly reported after chronic inhalation of GNPs for either 5 or 28 days, even at the highest dose and despite the persistence of materials.^[^ ^20^, ^21^ ^]^

Similar to GNPs, pulmonary exposure to GO sheets was also reported to induce acute inflammation in rodent lungs.^[^ ^22^, ^23^, ^24^ ^]^ Importantly and comparing different GO materials, Li et al. demonstrated that higher degree of oxidation correlated with higher level of lung inflammation.^[^ ^20^ ^]^ These results agree well with another study in which GO sheets exhibited higher inflammation levels than GNPs when the two materials were compared.^[^ ^22^ ^]^ In addition, when comparing GO and reduced GO (rGO) after single intratracheal administration, Bengston et al. reported that both materials were genotoxic in BAL immune cells but not in lung tissues.^[^ ^24^ ^]^ They also reported that rGO could cause chronic lung inflammation whereas GO‐induced inflammation was only acute.^[^ ^24^ ^]^ The importance of lateral dimensions was also highlighted in several studies that reported an increased inflammatory profile for larger materials (either GNPs or GO sheets).^[^ ^14^, ^25^, ^26^, ^27^, ^28^ ^]^ Although tissue recovery was observed in most of the aforementioned studies, large GO sheets were shown to cause long lasting lung injuries and fibrosis, owing to a reduced clearance from the alveolar region.^[^ ^25^, ^29^ ^]^ Equally, we have previously reported that the mechanisms involved in the adverse effects of GO are size‐dependent, with the number of molecular pathways indicative of adverse outcomes decreasing with the lateral dimensions of the GO sheets.^[^ ^27^ ^]^ Going further, we ascribed this size‐dependent activation of adverse outcome pathways in the lung to the slower tissue recovery caused by repeat exposure to micrometric GO compared to nanometric GO.^[^ ^28^, ^30^ ^]^

As highlighted above, inhalation of GBMs has raised several health concerns. It follows that proposing GBMs as the next platform for pulmonary drug delivery can be perceived as challenging. Nevertheless, GBMs could still be promising nanocarriers for the respiratory tract if the balance between benefits and risks is accurately and systematically evaluated. Indeed, GBMs offer a unique opportunity for enhanced targeted delivery to the alveolar space, owing to their distinctive biological properties. Namely, their deep lung penetration, their natural internalization in macrophages, their accumulation but at the same time continuous elimination from the lungs, and the overall rapid lung recovery reported in several if not most of the existing studies could all be deemed advantageous features.^[^ ^14^, ^19^ ^]^ Noticeably, targeting alveolar cells could be of interest in the context of lung infections by pathogens, such as mycobacteria that undergo intracellular replication in alveolar macrophages, or for cancer therapy, as cancer cells tend to grow in the alveolar cavity in many lung carcinomas.^[^ ^31^ ^]^ Once accumulated in macrophages, drugs carried by GBMs would be expected to be slowly released to nearby lung cells upon degradation of the GBM nanovectors.^[^ ^27^, ^32^ ^]^

To exploit this opportunity and address all concerns and remaining challenges, the in vivo fate and behavior of GBMs in relation to their biological impact on lungs will need to be systematically evaluated. Indeed, regulators would only approve a GBM‐based nanovector for pulmonary delivery if it is demonstrated that this vector is inert to the lungs and rapidly eliminated from the airways. In this respect, a thorough study of GBM fate over time is missing due to the difficulty of tracking GBMs in vivo, as they are made of carbon, the main element of organic matter. In addition, although previous studies have reported that GBMs can degrade in vitro^[^ ^33^ ^]^ and in tissues in vivo,^[^ ^27^, ^34^ ^]^ there is no definitive information explaining how GBMs are getting eliminated from the lungs. Both degradation by immune cells and clearance mediated by immune cell migration or lung fluid flows (interstitial and mucocilliary) could be responsible for GBM elimination from the airways and alveolar cavity. Moreover, due to the limited number of comparative studies between FLG and GO, it remains undetermined whether one or the other should be favored in the design of future lung delivery systems based on GBMs. In particular, how different physicochemical characteristics such as dimension, thickness, or carbon to oxygen ratio (or the combination of such features) may influence the degradation, elimination, and toxicological/biocompatibility profile of GBMs in the lungs remain poorly understood.

To address some of these knowledge gaps, we evaluated the pulmonary impact of four different GBMs over 28 days after single oropharyngeal aspiration in mice. Thin GO was compared to thicker FLG in order to understand the importance of the carbon to oxygen ratio as well as material thickness in these processes. Two lateral dimensions for each type of GBMs were also tested to evaluate the importance of size. A single exposure scenario was specifically chosen to investigate the potential of the different materials to degrade in cells over 28 days after exposure and track the administered materials in the lungs. We measured the cell loading and intracellular degradation of the selected GBMs using Raman microscopy, in all cells present in the alveolar space in vivo. Using this data set, we could also determine their kinetics of elimination from the alveolar space over the recovery period (from day 1 to 28). Finally, we examined the biological effects in respect to GBMs’ physicochemical characteristics and correlated the biological effects with material fate, in order to determine the combination of features leading to the best biocompatibility profile in the lungs.

## 2 Results and Discussion

### 2.1 Graphene‐Based Material Production and Characterization

We produced four different types of GBMs from graphite, following two well‐established methods previously reported by our groups. GO sheets (1–2 nm thick due to hydration) with either nanometric (SGO; average ≈60 nm) or micrometric (LGO; average ≈8 µm) lateral dimensions were synthetized by the Hummers’ method.^[^ ^35^ ^]^ FLG sheets (3–7 layers and thickness >5 nm) of nanometric (SFLG; average ≈200 nm) and micrometric (LFLG; average ≈1 µm) lateral dimensions were produced using riboflavin as an exfoliating agent.^[^ ^36^, ^37^ ^]^

**Figure 1:**
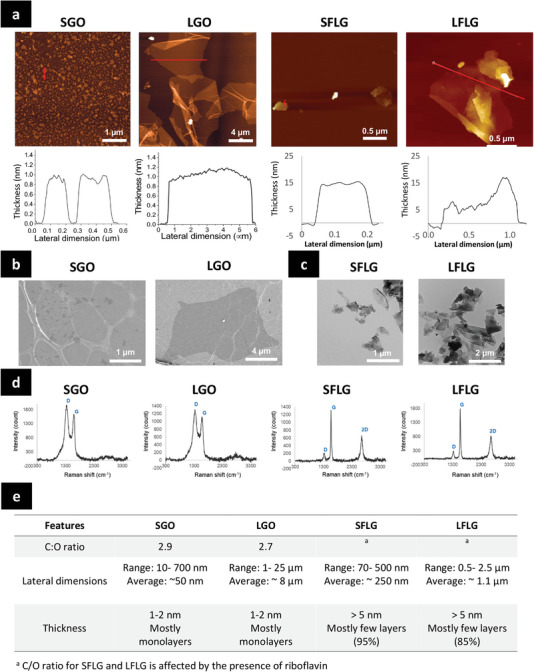
Structural and morphological characterization of the GBMs. a) Atomic force microscopy (AFM) images of SGO, LGO, and LFLG. b) Scanning electron microscopy (SEM) images of GOs. c) Transmission electron microscopy (TEM) images of FLGs. d) Raman spectra of the GBMs. e) Summary of GBMs physicochemical characteristics; C/O ratio was evaluated using X‐ray photoelectron spectroscopy (XPS), Lateral dimensions were evaluated using AFM (GOs and LFLG), SEM (GOs), TEM (FLGs) and optic microscopy (LGO). Thickness was evaluated using AFM (GOs and LFLG), Raman (FLGs) or TEM (FLGs).

The main physicochemical characteristics of the four materials are reported in **Figure** **1**a–e. The results demonstrated that for each category of GBMs, either GO or FLG, we achieved two distinct size distributions with minimal overlapping, allowing the comparison between different lateral dimensions. We also confirmed that GOs and FLGs had distinct C:O ratio (with GO being the more oxidized materials, irrespective of lateral dimensions) and distinct thickness (with FLG being the thicker materials with thickness going up to 15–20 nm in some part of the flakes as seen in Figure 1a, regardless of lateral dimensions). In order to perform a biological assessment without bias, we also confirmed that all four GBMs were endotoxin free using a method previously published.^[^ ^38^ ^]^

### 2.2 Immune Infiltration and Tissue Changes in Response to Graphene‐Based Materials Presence in Lungs

#### 2.2.1 Immune Infiltration in the Alveolar Space

**Figure 2:**
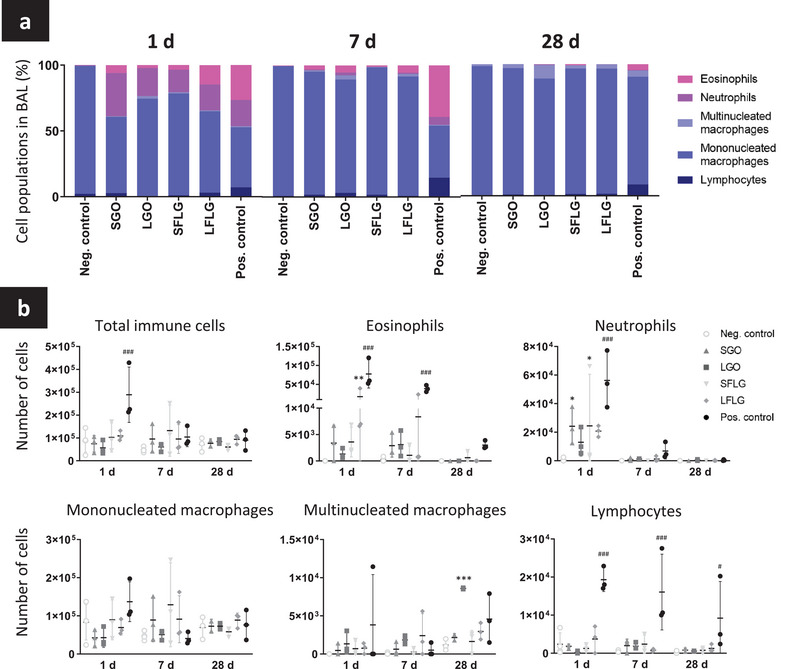
Variation of immune cell populations in the alveolar space. Mice were exposed by oropharyngeal aspiration to 30 µg of GBMs or controls (positive control: MWCNTs; negative control: water for injection). Alveolar cells were collected at day 1, 7, and 28, cyto‐spun on slides and then stained with colorimetric dyes for cell phenotyping. a) Percentage of each immune cell population identified. b) Total number of immune cells, eosinophils, neutrophils, mononucleated macrophages, multinucleated macrophages, and lymphocytes in BALF. Two‐way ANOVA followed by Tukey's multiple comparisons test were used to evaluate statistical differences in GBM number compared to the negative control. Statistical analysis performed separately for the GBMs (*p* < 0.05:^*^, *p* < 0.01:^**^, and *p* < 0.001:^***^) and for the positive control (*p* < 0.05:^#^, *p* < 0.01:^##^, and *p* < 0.001^:###^) (*n* = 3).

To assess the biological impact of the four different GBMs on lungs, we first evaluated the influx of immune cells in the alveolar space, using differential staining of cells contained in BAL fluids (**Figure** **2**a,b for numerical results, Figure S1, Supporting Information, for pictures). At day 1, we found a clear influx of neutrophils for the four tested GBMs compared to the vehicle control. The difference was statistically significant only for SGO and SFLG. Some eosinophils were also identified, but the influx was statistically significant only for LFLG. After 7 days, the number of neutrophils was back to normal for all conditions, but not for the positive control, namely MWCNTs (MWCNT‐7; Mitsui‐7), a well‐established nanomaterial causing inflammation, fibrosis and DNA damages.^[^ ^39^ ^]^ The number of eosinophils was also decreasing for all GBMs except LGO, which saw an increase at day 7 in comparison to day 1 in a similar manner to the positive control. Both neutrophil and eosinophil levels were back to normal at day 28 for all four GBMs, but not for the positive control MWCNTs that still presented noticeable eosinophils in the alveolar space, as expected for this nanomaterial causing an allergy‐like response.^[^ ^28^ ^]^

Importantly, lymphocytes were not significantly present in BAL fluids following exposure to any GBMs. This is in clear contrast with the MWCNTs that sustained a significant lymphocyte influx at each time point (Figure 2). Similarly, the number of mononucleated macrophages did not change after exposure to any of the four GBMs (Figure 2b). However, we observed a significant increase in the number of multinucleated macrophages for the LGO at day 28 compared to the vehicle control. Multinucleated macrophages are the product of macrophage fusion that occurs when foreign bodies or materials cannot be internalized and eliminated properly due to large dimension.^[^ ^40^ ^]^ Moreover, presence of multinucleated macrophages in lungs has been previously associated with frustrated phagocytosis and clearance issue following the inhalation of high aspect ratio materials (HARN) such as long MWCNTs.^[^ ^41^, ^42^ ^]^ Herein, only the largest material namely LGO (≈8 µm), but not LFLG (≈1 µm), led to the formation of multinucleated macrophages, suggesting that only this material could not be cleared properly from the lungs, in agreement with the HARN frustrated phagocytosis paradigm and its associated 5 µm threshold. ^[^ ^18^, ^41^, ^42^ ^]^

#### 2.2.2 Biological Response in Lung Tissues

**Figure 3:**
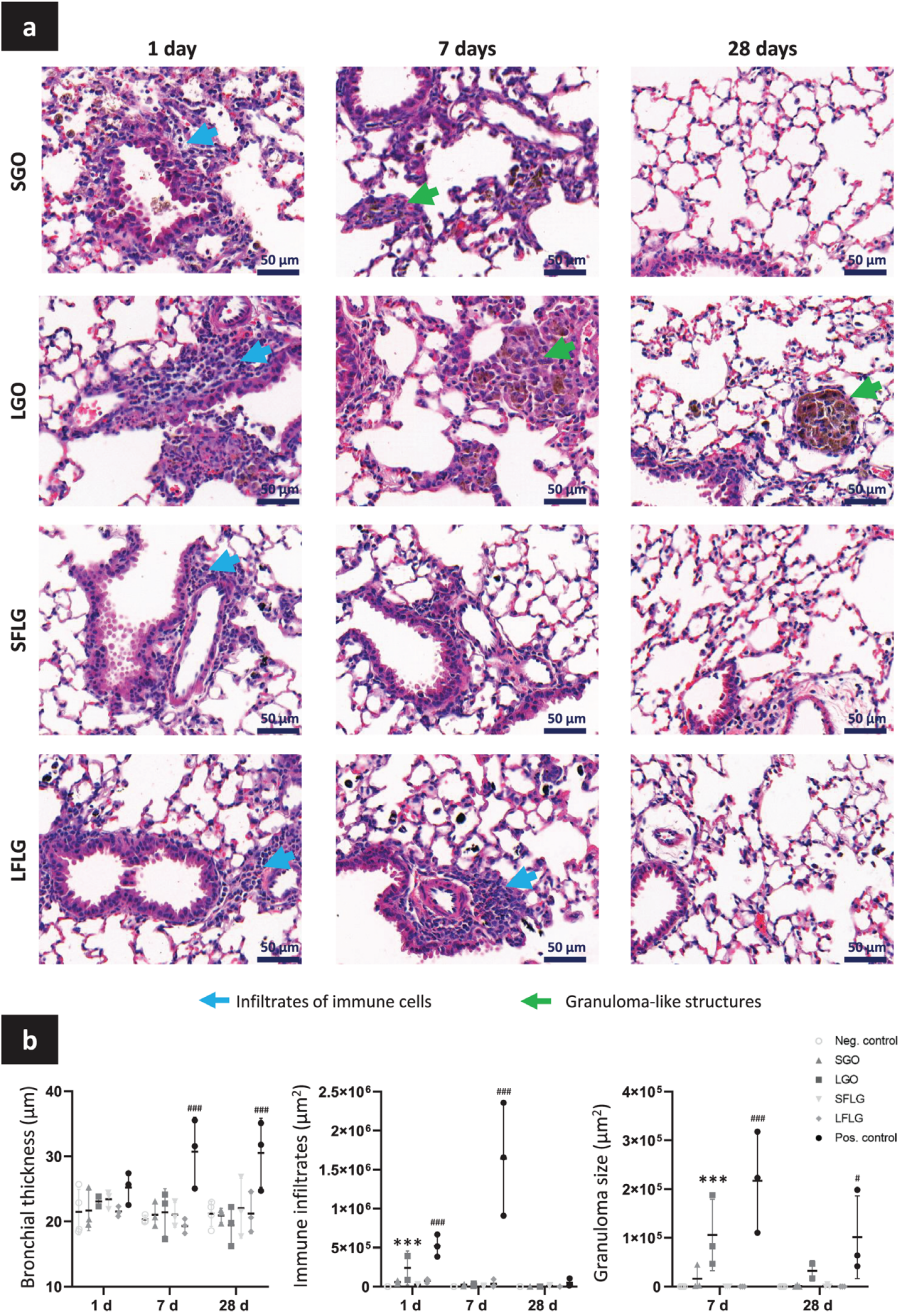
Transient immune structures attributable to GBM presence in the lungs. Mice were exposed by oropharyngeal aspiration to 30 µg of GBMs or controls (Positive control: MWCNTs; Negative control: water for injection). Collected left lungs were inflated with formalin, embedded in paraffin and then processed. a) Lung sections (5 µm) were stained in Haematoxylin‐Eosin for histopathological analysis. Immune infiltrates (blue arrow) and granulomatous‐like structures (green arrow) were identified. b) Sizes of immune infiltrates and granulomatous‐like structures and bronchial thickness were recorded and comparisons were performed between the different GBMs and controls. Two‐way ANOVA followed by Tukey's multiple comparisons test were used to evaluate statistical differences in GBM number compared to the negative control. Statistical analysis performed separately for the GBM (*p* < 0.05:^*^, *p* < 0.01:^**^, and *p* < 0.001:^***^) and for the positive control (*p* < 0.05:^#^, *p* < 0.01:^##^, and *p* < 0.001:^###^) (*n* = 3).

We then determined how the lung tissue responded to GBM presence, using histopathological analysis of lung sections (**Figure** **3**a,b; Figure S2, Supporting Information). First, the bronchial thickness was evaluated to assess the impact of GBMs on the bronchial tract. No variation in bronchial thickness was found for any of the GBMs compared to the negative control (water for injection). Only the positive control (MWCNTs; MWCNT‐7) induced a significant increase in bronchial thickness at day 7 and 28 (Figure 3b).

Second, we assessed the presence of immune cell infiltrates in the lung sections. Cell infiltrates were identified for all materials (Figure 3a; Figure S2, Supporting Information) at day 1, but significant only for LGO and positive control. This is in agreement with the BAL analysis showing immune cell recruitment (Figure 2a). However, we noted some variations between the different GBMs tested. Exposure to SGO or any of the two FLGs led to a diffuse infiltration of granulocytes into the lungs. In contrast, LGO (and the positive control MWCNTs) induced a more localized and distinctive infiltration of granulocytes around bronchia and blood vessels. This was materialized by an increased number and size of immune cell infiltrates at day 1 for LGO in comparison to other GBMs (Figure 3b). Yet, the LGO‐induced infiltrates were still in lower amount compared to the MWCNT positive control. The more diffuse immune responses found at day 1 for SGO and both types of FLGs could be attributed to a better distribution of these materials across the lungs, including in alveoli, as suggested by the Raman imaging analysis of lung sections (Figure S3, Supporting Information). In comparison, LGO sheets formed large material agglomerates localized primarily next to bronchia (brown agglomerates in H&E images, Figure S2, Supporting Information), suggesting that their larger dimensions may hinder their transport from the upper airways to the alveolar space and favor material agglomeration. At day 7 and 28, no significant immune cell infiltrates were found for any of the GBMs (Figure 3b). This rapid lung recovery highlighted that the four GBMs had a moderate impact on lungs, especially when compared to the persisting inflammatory effects of MWCNTs at day 28.

Besides immune cell infiltrates, we also noted the formation of granuloma‐like structures, which were more apparent for LGO compared to other GBMs (Figure 3a; Figure S2, Supporting Information). Interestingly, high amounts of materials were detected in these structures using Raman microscopy (Figure S3, Supporting Information). Moreover, some of these structures were still present at day 28 for LGO, though fewer compared to day 7, suggesting that localized accumulation and persistence of materials may be associated to the formation of these granuloma‐like structures. The greater number of granuloma‐like structures found in LGO exposed animals in comparison to the other GBMs may be explained by an impaired clearance of LGO materials from the lungs, as evidenced by the longer lasting presence of LGO in lung sections analyzed by Raman spectroscopy (Figure S3, Supporting Information) compared to the other three GBMs. We theorized that these granulomatous structures are made of macrophages that fused and formed cell clusters in order to eliminate the LGO agglomerates more efficiently.^[^ ^43^ ^]^ Similar observations have been made for pathogens such as mycobacterium or other foreign bodies that find their way to the alveolar space.^[^ ^43^ ^]^ In addition, the presence of granuloma‐like structures attached to the alveolar or airway walls is in agreement with the presence of multinucleated macrophages in BAL fluids (Figure 2a,b; Figure S1, Supporting Information), further suggesting that LGO sheets suffer from a clearance issue, likely due to their larger dimensions. Importantly, despite their ostensible presence in LGO exposed lungs, the number of granulomatous structures was still measurably lower than after exposure to the positive control MWCNTs.

Taken together, these results demonstrated that GBMs caused an acute immune response in the lungs, as shown previously in other studies, including ours.^[^ ^16^, ^17^, ^18^, ^22^, ^24^, ^44^ ^]^ Noticeably, the recovery of the lungs from this acute inflammation was rapid, even after LGO exposure. This is in sharp contrast to the response found for the positive control, namely long and rigid MWCNTs, which have been repeatedly reported to induce chronic inflammation, fibrosis, tissue remodeling, and even cancer.^[^ ^41^, ^42^ ^]^ However, the presence of multinucleated macrophages, granuloma‐like structures, and persistent material agglomerates in LGO‐treated mouse lungs warrants further investigations, in particular to assess the long‐term fate and possible consequences of these biopersistent materials in the lungs.

### 2.3 Graphene‐Based Material Alveolar Accumulation

Most of the biological outcomes reported above for LGO could be associated to a greater persistence in the alveolar space of these materials compared to the other three GBMs. To assess alveolar persistence, we used Raman imaging to measure GBM presence at each time point and evaluate the evolution of materials’ presence over time as indicator of persistence, clearance, and/or degradation (Figure S3, Supporting Information), as previously reported.^[^ ^27^, ^45^, ^46^ ^]^ However, this method does not provide a quantitative measurement of the amount of GBMs present in the whole lungs. Indeed, only few lung sections were scanned, and within a given section, only specific regions of interest (ROI) were imaged. Such analysis is therefore only indicative of the relative amount of GBMs and its variation over time (i.e., a decrease of Raman signature with time suggesting a reduction of GBM lung burden). Moreover, Raman analysis of lung sections is not directly providing cell‐specific measurement. To overcome this limitation, we decided to image by Raman spectroscopy the alveolar phagocytes isolated from BAL fluids, as previously reported.^[^ ^28^ ^]^ Alveolar phagocytes (tissue resident and recruited upon inflammation) are known to internalize various nanomaterials after pulmonary exposure. Furthermore, once nanomaterials have reached the alveolar space, they are expected to remain in this compartment for some time, especially if not cleared rapidly.^[^ ^18^, ^19^ ^]^ Therefore, we reasoned that evaluating GBM loading in BAL phagocytes at each time point and over time in a semi‐quantitative way would represent a valuable approach to assessing the fate of GBMs (i.e., persistence, degradation, and clearance) and would be complementary to the qualitative assessment done at organ level on tissue sections.

#### 2.3.1 Graphene‐Based Material Accumulation in Alveolar Phagocytes

**Figure 4:**
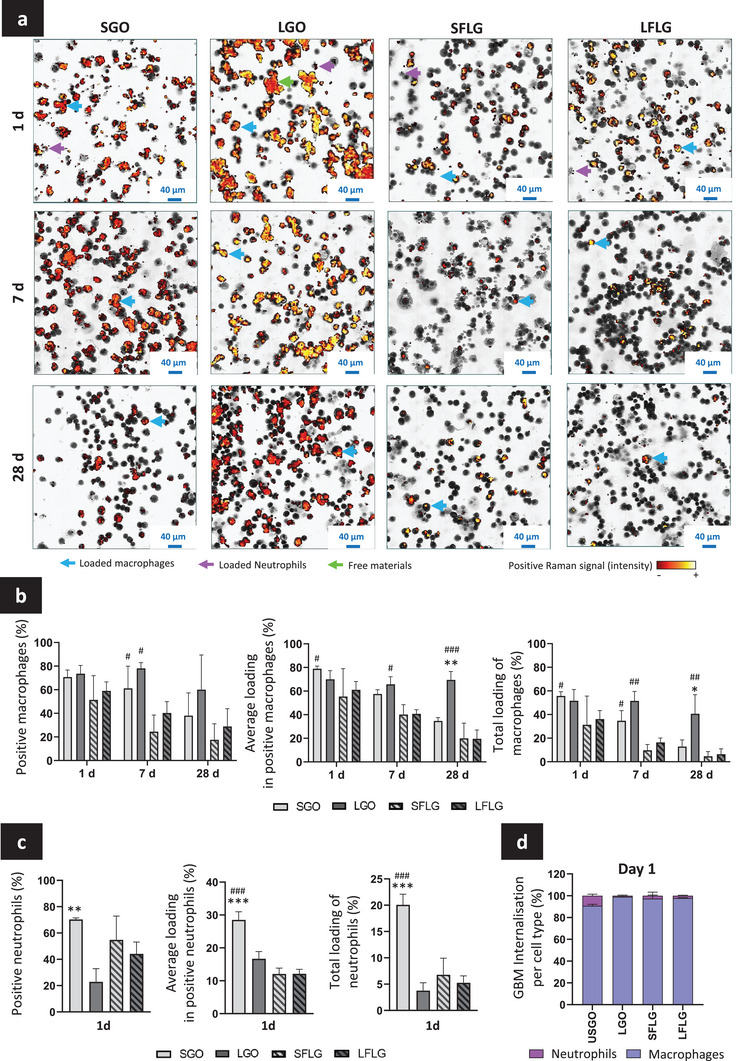
Evaluation of GBM presence in alveolar phagocytes. BAL fluids were collected and alveolar cells were cyto‐spun on slides. Fixed cells were first analyzed by Raman scanning microscopy and second stained with colorimetric dyes. Then, materials' signal (G bands for GO and FLG) and cell phenotyping images were overlapped to evaluate GBM internalization in alveolar phagocytes. a) Raman signal in alveolar cells at day 1, 7, and 28 after exposure. b) Internalization of GBMs in macrophages at day 1, 7, and 28. c) Internalization of GBMs in neutrophils at day 1. d) Proportions of internalized GBMs in macrophages and neutrophils at day 1. Two‐way ANOVA (for macrophages) or one way ANOVA (for neutrophils) followed by Tukey's multiple comparisons test were used to evaluate, at each time‐point, statistical differences between GBMs respective large and small sizes (USGO vs LGO; SFLG vs LFLG: *p* < 0.05:^*^, *p* < 0.01:^**^, and *p* < 0.001:^***^) or chemical characteristics (SGO vs SFLG; LGO vs LFLG: *p* < 0.05:^#^, *p* < 0.01:^##^, and *p* < 0.001:^###^) (*n* = 3).

First, BAL fluids were collected from GBM exposed lungs at 1, 7, and 28 days after oropharyngeal aspiration. The BAL cell suspensions were carefully homogenized to achieve a good spatial distribution of the isolated cells on the glass slide during cyto‐spinning. This step was important to ensure that the image of these cells would be representative of the whole alveolar cell population present in the airways at the considered time point. After fixation, cyto‐spun BAL cells were first imaged by Raman to evaluate the presence of GBMs. GBM presence was determined using the specific Raman signatures of the four fully characterized materials tested here (i.e., D and G bands for GO; G and 2D bands for FLG; Figure 1d). Following Raman imaging, the same glass slides were subsequently stained using Diff‐Quick stains and imaged by bright‐field optical microscopy in order to phenotype the different immune cells present in BAL fluids. Finally, Raman maps (G band only) and bright‐field images were overlapped (**Figure** **4**a) to correlate the cell identity with GBM presence. Using this approach, we identified that only macrophages (blue arrow) and neutrophils (purple arrow) had internalized materials, since we recorded positive Raman signatures only in these two cell types. Importantly, we did not identify GBMs in any of the other cells present in BAL fluids (i.e., eosinophils, lymphocyte, and detached bronchial cells). These results are in agreement with the expected phagocytic ability of neutrophils and macrophages. For each GBM, we then determined the number of macrophages or neutrophils that contained a positive Raman signature (i.e., how many cell in each category contained GBMs) and evaluated the average material loading per positive cell (i.e., how much GBMs each loaded cell contained). Subsequently, we calculated the total material loading per cell type, including both positive and negative cells (i.e., how much GBMs is present in either macrophages or neutrophils when the whole BAL cell count is taken into consideration; Figure 4b,c), as previously reported.^[^ ^28^ ^]^ Using the three parameters all expressed as percentage (i.e., number of positive cells, average loading per positive cell, total material loading per cell type; see further details about how these different values were calculated in the experimental section), we finally compared the material loading in macrophages or neutrophils over time and between the different types of GBM used.

#### 2.3.2 GBM Accumulation in Alveolar Macrophages

At day 1, 7, and 28 after exposure, there was a higher number of Raman positive pixels per surface area of a considered cell for GOs compared to FLGs (Figure 4b). This result suggests that there was a greater material loading per BAL macrophage for GOs than for FLGs. At day 1, this trend was found for the two GOs in comparison to the two FLGs, but appeared statistically significant only when comparing SGO to SFLG. At day 7, a significant increase was measured for both GOs in comparison to both FLGs. After 28 days, the difference in cell loading was significant only for LGO, but not for SGO, in comparison to the two FLGs. All time points considered, these results demonstrated the accumulation of all types of GBMs in macrophages, but with relatively more cell loading for GOs compared to FLGs. The differences in material thickness between GOs and FLGs could explain the variation in cell loading. Here, both SGO and LGO were made mostly of monolayers, while both types of FLG were mostly few layered (Figure 1a,e). Thus, for the same mass of materials administered (assuming that the delivered dose to the lungs is identical between GOs and FLGs), the number of particles per lung surface area, hence the total lung surface area potentially covered by FLGs, was expected to be less in comparison to GOs. At the cell level, this also implies that for the same mass of materials delivered to macrophages, GOs would occupy a larger space (surface area/volume) within the cell than would FLGs. These results are important because higher macrophage loading has often been associated with higher biological responses.^[^ ^47^ ^]^

When comparing the material loading between days 1 and 7, we measured a clear reduction in FLG signal in BAL macrophages, while the decrease in GO signal was less pronounced (Figure 4b). After 28 days, the four GBMs were still present in BAL macrophages (Figure 4b), but we noted a clear decrease in cell loading for SGO, SFLG, and LFLG in comparison to day 1 or 7. For LGO, strong Raman signal indicative of high material loading was still detectable in BAL macrophages after 28 days. Interestingly, when comparing the two FLGs (small versus large), there was no difference in the cell loading at any time point. In contrast, there was a clear difference between SGO and LGO in the evolution of the material contained in macrophages over the 28‐day recovery period. The total material loading of the macrophage population was the parameter showing the most striking difference between the two GOs, with both the number of positive macrophages and the average loading per positive macrophage decreasing over time for SGO but not for LGO. This last result clearly demonstrated that the larger material (≈8 µm) had greater persistence in lungs compared to the other GBMs, suggesting the existence of a size‐threshold for GBMs’ persistence in the lungs, as reported previously for high aspect ratio nanomaterials, such as fibers.^[^ ^48^ ^]^

Overall, these set of results underline the importance of material thickness and lateral dimension in the internalization and retention of GBMs in macrophages. Internalization of GBMs by macrophages has been explored before in several in vitro or in vivo studies.^[^ ^34^, ^49^, ^50^, ^51^, ^52^ ^]^ Noticeably, some of us highlighted the benefit of using Raman microscopy to estimate in vitro the accumulation of GO with varying lateral dimensions in macrophage cell lines.^[^ ^49^ ^]^ In this study, there were more macrophages positive for the smaller GO (90%) than positive for the larger GO (60%) after 24 h of exposure, suggesting that smaller materials have greater internalization prospect than larger materials. These results (obtained with a GO of different origin than the one used here and that possessed a smaller lateral dimension than the large GO with an average of 1.32 µm) contrast with our present data showing that at day 1 there was little to no difference between large and small materials in terms of cell loading, irrespective of the GBM type considered (Figure 4b). Nevertheless, comparing in vitro to in vivo results is difficult as materials behave very differently in cell culture medium and in the complex environment of the airways. Sedimentation and agglomeration are often neglected variables in vitro, although they are critical parameters that could affect material deposition and internalization by cells.^[^ ^53^ ^]^ Moreover, these parameters are material specific, hence would vary with different GO production. In an in vivo scenario, other parameters would also need to be factored in, including a different type of biomolecule coronation, as well as a more dynamic environment. These parameters specific to the in vivo environment may explain why macrophage uptake was not size‐dependent at the earliest time point in the present study.

#### 2.3.3 GBM Accumulation in Neutrophils

Neutrophils are typically recruited from the blood to the respiratory tract upon induction of an innate immune response following lung exposure to airborne particles, in order to help reducing the lung burden.^[^ ^54^ ^]^ Besides the interaction with BAL macrophages, the four GBMs could hence interact with or accumulate in recruited neutrophils at day 1 (Figure 4a,c). Since neutrophils were no longer present in BAL fluids beyond day 1, we could only estimate GBM accumulation in neutrophils at this time point (Figure 2). Interestingly, BAL neutrophils had accumulated more SGO than any other tested materials (Figure 4c), with ≈70% of the recruited neutrophils demonstrating a positive Raman signal for SGO. In addition, all three measured parameters were statistically significant for SGO compared to LGO. There was also a significant increase in the average material loading and total neutrophil loading for SGO compared to the two FLGs. Comparing the two FLGs, the difference in lateral dimension had no significant impact on the cell loading, despite a slightly higher accumulation for SFLG compared to LFLG. These results highlighted again that lateral dimension is the leading feature in comparison to thickness or C:O ratio for GBM accumulation, here in neutrophils.

The presence of nanomaterials in neutrophils, including GBMs, has been reported before in several studies in vitro^[^ ^55^ ^]^ and in blood samples.^[^ ^54^, ^56^ ^]^ In these studies, the interaction of neutrophils with nanomaterials was shown to induce various downstream effects, from their activation to the induction of apoptosis, through inhibition of apoptosis. Moreover, it has also been reported that neutrophils could reverse migrate to lymph nodes, blood vessels, and even the bone marrow.^[^ ^57^ ^]^ Since we found here a greater affinity of the recruited neutrophils for SGO, both the further biological implications of this accumulation/interaction and the long‐term fate of SGO relocated to secondary organs via phagocyte reverse migration should be the focus of future investigations. In particular, it would be important to assess in detail how much GBMs relocate from the lungs to secondary organs and the mechanisms (i.e., fluid drainage or cell‐mediated migration) leading to this translocation.

#### 2.3.4 Comparing GBM Accumulation in Macrophages and Neutrophils

Neutrophils and macrophages are both phagocytic cells. However, their internalization capacity is different, as confirmed by our direct comparison between the two cell types for their respective average material loading per cell and total loading per cell type at day 1 (Figure 4b,c). When comparing the proportion of materials internalized at this time point in these two cell populations, we found that macrophages had a greater cell loading than neutrophils, regardless of the material considered (Figure 4d). This difference can be ascribed not only to the greater number of macrophages in the alveolar space but also to their larger size and uptake capacity in comparison to neutrophils (Figure 2a). Considering that neutrophils were not present in the BAL fluid beyond day 1, we inferred that macrophages were across all time points the main cell type involved in GBM elimination from the alveolar space.

Unexpectedly, alongside the specific patterns of internalization described for LGO at day 1 in either macrophages or neutrophils, we also found a substantial number of free LGO sheets (green arrow) that were not present in any phagocyte (Figure 4a). However, this was not the case for SGO or the two FLG materials, suggesting that the combined alveolar phagocytes (macrophages and neutrophils) were able to engulf all materials present in the alveolar space in the case of SGO, SFLG, and LFLG, but not LGO. The larger lateral dimension of LGO sheets leading to a lower LGO cell loading in both neutrophils and macrophages in comparison to the other GBMs (Figure 4d) could explain the presence of free LGO sheets at day 1 (Figure 4a). Indeed, we reasoned that macrophages present at day 1 in the alveolar space may have reached their maximum loading capacity, and while the recruited neutrophils were able to engulf or interact with the remaining materials in the case of SGO, SFLG, and LFLG, they could not support the macrophages and engulf all remaining materials in the case of LGO. On the other hand, the presence of free LGO materials at day 1 may explain why LGO were identified in macrophages for a longer period of time, barely decreasing from day 1 to 28 (Figure 4b), if we theorized that new macrophages were recruited to engulf the remaining LGO sheets. Future studies should aim at interrogating the turnover and fate of LGO laden macrophages to reveal whether the laden macrophages present at day 1 are the same as those present at day 28 and reveal the fate of the free LGO sheets in the lungs and beyond.

Taken together, these results highlighted that lateral dimension is a dominant physicochemical feature with respect to the accumulation of GBMs in alveolar phagocytes. Since we demonstrated that most of the free LGO sheets were internalized in macrophages by day 7 (Figure 4a,b), it was unclear why a high quantity of materials were still present in macrophages at day 28. We hypothesized that this persistence may be due to impaired degradation of the LGO materials in macrophages and therefore decided to further assess the level of GBM degradation in alveolar phagocytes.

### 2.4 Alveolar Clearance and Degradation of Graphene‐Based Materials

**Figure 5:**
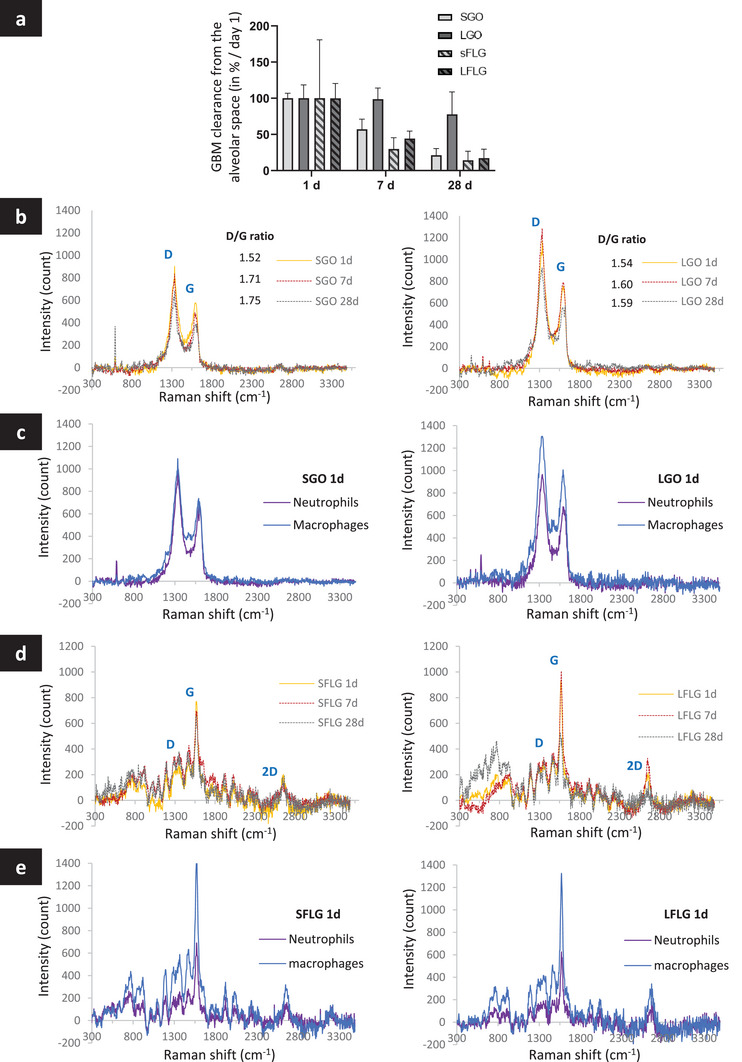
GBM clearance and intracellular degradation over time in lung phagocytes. Mice were exposed to 30 µg of GBMs and BALF were collected at day 1, 7, and 28. Alveolar cells were scanned by Raman and stained for cell phenotyping to evaluate the clearance and degradation of GBM. a) GBM clearance over time. b) Intracellular degradation of GO over time in alveolar phagocytes and c) at day 1 in neutrophils compared to macrophages. d) Intracellular degradation of FLG over time in alveolar phagocytes and e) at day 1 in neutrophils compared to macrophages. Raman signals were refined by removing the biological background. To evaluate GBM degradation, D/G ratio was calculated for each animal and variation of positive Raman signatures were evaluated. For each material and time‐point, the corresponding average intensities and D/G ratio of 3 animals are presented in the graphs (30–3000 positive spectra for GBM signature were recorded for each animal, depending on the amount of materials detected).

Considering the natural ability of alveolar phagocytes to remove, process, and degrade foreign materials including carbon‐based materials,^[^ ^58^, ^59^ ^]^ we evaluated the level of GBM alveolar clearance and degradation in both macrophages and neutrophils. To this end, we used Raman‐based values, as calculated above, and analyzed the spectra of the Raman maps (see Experimental Section for further details) (**Figure** **5**a).

#### 2.4.1 Alveolar Clearance of GBMs

To estimate the overall alveolar clearance of GBMs over time (i.e., how much material disappears from the alveolar space due to engulfment in alveolar phagocytes), we used the values of total material loading in macrophages and neutrophils calculated at day 1, 7, and 28 (Figure 4b,c; see Experimental Section for further details). At day 1, we were able to use the combination of total material loading values obtained for macrophages and neutrophils. However, at day 7 and 28, only the macrophage value was available since neutrophils were no longer present in the alveolar space (Figure 2a). Day 1 constituted the starting point for these assessments (i.e., maximum material loading value for each GBM equals to 100%, clearance equals to 0%). Days 7 and 28 were the only two time points at which clearance from the alveolar space could be determined in comparison to day 1 (with alveolar clearance expressed in percentage as = 100% – % of remaining materials) (**Figure** **5**a). On days 7 and 28, compared to day 1, we found clear and progressive clearance for three of the GBMs, namely SGO, SFLG, and LFLG (Figure 5a). The alveolar clearance appeared to be slightly faster for the two FLGs (SFLG: ≈30% remaining at day 7 and only ≈14% at day 28; LFLG: ≈44% remaining at day 7 and only ≈17% at day 28) compared to SGO (≈57% remaining at day 7 and only ≈21% at day 28). In contrast, LGO clearance from the alveolar space was significantly slower, with no apparent elimination found between days 1 and 7 (≈98% remaining, clearance of 2%), and still ≈80% of the materials remaining after 28 days, resulting in only a 20% alveolar clearance compared to day 1 (Figure 5a). These results not only confirm the persistence of LGO in macrophages and hence in the lungs but also highlight the role of lateral dimensions, material thickness, and carbon to oxygen ratio in the speed of material clearance from the alveolar space.

#### 2.4.2 GBM Degradation in Alveolar Phagocytes

To complement the alveolar clearance, we then evaluated the level of intracellular elimination (partial or complete degradation) of GBMs in BAL cells (Figure 5b–e; Figure S3a,b, Supporting Information). For this purpose, we used the decreases in Raman peak (D and G bands) intensities and changes in Raman peak intensity ratios as indicators of such material alteration in cells. A clear change in intracellular GBM signature was found between days 1 and 28, as demonstrated by a decrease in the overall Raman intensity for all four GBMs (Figure 5b,d). In addition, both an increase in the *I*_D_/*I*_G_ ratio and an alteration of the G peak were recorded for the two GO materials (Figure 5b). These variations in peak forms and intensities are known indicators of degradation or biotransformation in biological systems for carbon‐based materials, as previously reported by us and others.^[^ ^27^, ^33^, ^34^, ^45^, ^60^, ^61^ ^]^ Remarkably, we noted a faster alteration of the Raman spectra that we attributed to degradation for the smaller materials in comparison to their larger counterparts. This was clearly illustrated with the GO materials, with a more pronounced and faster increase in the *I*_D_/*I*_G_ ratio between days 1 and 7 for SGO (from 1.52 to 1.71) compared to LGO (from 1.54 to 1.60) (Figure 5b; Figure S3, Supporting Information). Moreover, there was no increase in the *I*_D_/*I*_G_ ratio between days 7 and 28 for LGO, whereas a slight increase was found for SGO. Interestingly, when comparing phagocytic cell types at day 1, we found a more obvious decrease in Raman signal intensity in neutrophils than in macrophages for all the GBMs tested (Figure 5c,e). This cell type difference suggests a faster degradation in neutrophils than in macrophages and underlines the important role of neutrophils in the elimination and degradation of GBMs from the lungs during the early phase response. Further evaluation would be necessary to identify potential biodegradation by‐products in macrophages and neutrophils.

#### 2.4.3 Impact of GBM Physicochemical Characteristics on their Degradation and Elimination from the Lungs

The findings of cell‐mediated in vivo degradation of GBMs (via macrophages and neutrophils) are in agreement with previous studies on the ability of various GBMs to be biodegraded. For instance, Girish et al. demonstrated for the first time in vivo the important role of tissue‐resident macrophages in the long‐term degradation of carboxyl‐functionalized graphene materials after intravenous injection.^[^ ^34^ ^]^ Using Raman confocal microscopy, they evaluated the material degradation for up to 3 months after IV injection in four different tissues of material accumulation and for up to 7 days in macrophage cell lines to confirm the role of macrophages in the degradation. The highest level of degradation was found in the spleen, the smallest in the lungs, with degradation always starting from the edges of the material agglomerates. They confirmed that macrophage cell lines were able to degrade materials to the same extent as the degradation found in vivo. Similar to the present study, they reported an increase in the *I*_D_/*I*_G_ ratio and a decrease in Raman intensity with time. In previous in vivo studies, we have also demonstrated that GO sheets accumulated in the spleen after IV injection were getting degraded over a 270‐day period in the marginal zone macrophages, or GO sheets present in the brain after intranasal administration were degrading over 28 days in the resident microglia and perivascular macrophages.^[^ ^45^, ^46^ ^]^

Taken together, these different studies agree with our findings and confirm that recruited or resident macrophages, wherever they are located in the lungs, blood, or other organs, are essential cells in the long‐term entrapment and/or elimination of GBMs owing to their phagocytic and degradation capacities. Moreover, our present study also stresses the key role of neutrophils in this cell‐mediated degradation/elimination process, especially during the acute phase response, when neutrophils are recruited to the site of inflammation. In fact, neutrophils were shown here to be more efficient at degrading GBMs compared to macrophages, despite their lower phagocytic ability. These results are in agreement with previous studies suggesting that macrophages may be less potent than neutrophils at degrading GBMs in vivo due to their reduced enzyme‐based oxidative degradation capacity.^[^ ^62^ ^]^ Overall, our results confirmed that neutrophils are better at degrading materials, whereas macrophages are better at clearing materials, even if both cell types have degradation and clearance abilities.

In respect to degradation mechanisms, a series of studies have described the different processes leading to the biodegradation of carbon nanomaterials, including GBMs.^[^ ^63^ ^]^ Oxidative degradation via the enzymatic activity of peroxidases, such as myeloperoxidase (MPO), is one of them and was demonstrated both in vitro and in vivo.^[^ ^63^, ^64^ ^]^ Using an acellular human MPO model from neutrophils, Kurapati et al. have reported that GO degradation was dependent on the level of material agglomeration, with well‐dispersed materials degrading better.^[^ ^61^ ^]^ Degradation was also achieved when single‐ and few‐layer graphene were exposed to human MPO, and was evidenced by a loss of crystalline and geometrical structure and an increase in *I*_D_/*I*_G_ ratio with time.^[^ ^33^ ^]^ In the same study, the two materials were exposed to activated human primary neutrophils and shown to degrade within 5 days. Moreover, both a decrease in G band intensity and an increase in *I*_D_/*I*_G_ ratio were evidences that an MPO‐mediated oxidative degradation caused by the neutrophil degranulation (and production of neutrophil extracellular traps (NETs)) had occurred.^[^ ^33^ ^]^ Using the same concept, the degradability of large and small GOs by activated primary neutrophils was demonstrated by a fast decrease in Raman intensity, with D and G bands significantly reducing after 3 and 6 h.^[^ ^60^ ^]^ Interestingly, MPO is produced in large quantity by activated neutrophils and to a lesser extent by macrophages. This difference in MPO expression capacity between neutrophils and macrophages would therefore explain the results reported here, with neutrophils degrading GBMs faster than macrophages. This capacity difference also implies that neutrophils may be more important than macrophages in the early phase of the response to nanomaterial exposure toward eliminating as much materials as possible to reduce the lung burden.^[^ ^65^ ^]^ It follows that materials that are not taken care of efficiently by the recruited neutrophils will be left to the attention of macrophages, which have a lower degradation capacity but greater phagocytic ability.

As a consequence, the lung persistence of LGO sheets found here could be explained by a combination of events. First, there is a reduced accumulation of LGO sheets in both neutrophils and macrophages and an incomplete internalization in macrophages at day 1 due to their larger lateral dimensions (LGO sheets being on average ≈8 µm, while the other materials, including LFLG, are below 1 µm). This suggests that there may be a size threshold for efficient internalization and elimination of GBMs by lung phagocytes, with GBM sheets above 5 to 10 µm inducing frustrated phagocytosis in macrophages and precluding/reducing internalization by recruited neutrophils, as previously reported for HARNs such as nanotubes. ^[^ ^48^ ^]^ Second, LGO sheets engulfed in neutrophils and macrophages are undergoing slower intracellular degradation compared to the other GBMs. In turn, this slower degradation explains their slower alveolar clearance (Figure 5a) and the persistence of a strong Raman signal in BAL macrophages at day 28 (Figure 4a,b). Future work should therefore aim at elucidating which molecular pathways in phagocytes may be affected by LGO to explain the apparent reduced degradation capacity of phagocytes that have internalized these materials.

Taken together, all the present findings point toward a link between the extent of GBM degradation (elimination/persistence) and the degree of adverse effects, with LGO materials causing the greatest impact among the four GBMs due to their reduced degradation and greater persistence. Similar inferences have been reached in previous works on carbon nanotubes, in which inhibition of nanomaterial degradation was associated with more adverse outcomes than when the natural degradation capacity was able to mitigate/reduce the effects of material exposure.^[^ ^62^, ^66^, ^67^ ^]^ While we noticed that LGO had a lower impact than MWCNTs, the induction of multinucleated macrophages, granuloma‐like structures, and persistent material agglomerates still present at day 28 is calling for further investigations about the long‐term impact of such persistence (i.e., beyond 3 or 6 months after exposure).

## 3 Conclusion

This comparative study between FLG and GO aimed to identify the physicochemical characteristics suitable for the design of safe pulmonary nanovectors based on GBMs. To this end, we investigated the lung tolerability, fate, and elimination profiles of four different GBMs (two graphene oxides and two few‐layer graphene; small and large) after single pulmonary administration in mice.

Compared to a reference material such as MWCNT‐7, none of the GBMs induced a strong immune response in the lungs, despite a transient influx of neutrophils and eosinophils, but with no recruitment of lymphocytes. However, exposure to LGO caused a focal immune response, with formation of granuloma‐like structures and presence of multinucleated macrophages, both of which were indicators of frustrated phagocytosis, often associated to impaired elimination and bio‐persistence of materials.

To assess the extent of material elimination from the lungs over time, we therefore evaluated by Raman microscopy the fate of GBMs both across the lungs and in the alveolar space over 28 days after exposure. While all the materials tested (SGO, LGO, SFLG, and LFLG) were present in the lungs at day 1, only LGO showed extended persistence at day 28. In the alveolar space, all tested GBMs were found in both macrophages and neutrophils, but not in other cells present in BAL fluids. Interestingly, GO cell loading appeared higher than FLG cell loading after 1 day, underlining that for the same dose more alveolar phagocytes could be targeted using GO. We attributed this difference to a difference in thickness between the two materials. FLGs being thicker occupied a smaller cell volume than GOs for the same mass of material administered. Furthermore, we revealed that recruited neutrophils at day 1 demonstrated a greater affinity for small materials and in particular SGO (the smallest and thinnest materials of all) compared to other GBMs. Yet, more GBMs in total amount were found in macrophages compared to neutrophils, underlining the dominant role of macrophages in the isolation and subsequent elimination or persistence of GBMs over time, especially during the inflammation resolution phase.

In respect to degradation and removal from the airways, SGO, SFLG, and LFLG were eliminated faster than LGO, the largest materials used in this study, as evidenced by a slower alveolar cell mediated clearance over time for this material. Importantly, our results highlighted the essential role of neutrophils in the early phase of material elimination and demonstrated that degradation of GBMs by neutrophils was size‐dependent. At day1, we showed a stronger intracellular degradation in neutrophils than in macrophages, which combined with the greater affinity of neutrophils for small sheets emphasized their crucial role in the elimination of small materials from the lungs at early time point. At later time points, the efficient clearance of SGO, SFLG, and LFLG from the lungs was associated to a clear intracellular degradation in macrophages. Conversely, the poor clearance of LGO was associated to an impaired degradation in macrophages over time, revealing that efficient degradation in macrophages is crucial for a controlled elimination from the lungs. Taken together, the degradation results suggested that small materials below 1 µm were degraded and eliminated faster and more efficiently than large materials above 5 µm, hence explaining the lung bio‐persistence of LGO compared to the other GBMs.

In summary, the present findings stressed the importance of each physicochemical feature such as lateral dimension, thickness, or carbon to oxygen ratio in the design of safe GBMs in respect to lungs. They demonstrate that features that do not favor degradation and elimination lead to material bio‐persistence, which in turn triggers biological responses often associated with long‐term adverse effects. Among the different GBMs tested here, LGO sheets showed the least biocompatible profile whereas SGO, SFLG, and LFLG may represent suitable options for pulmonary drug delivery systems. In particular, SGO demonstrated a limited innate response and a very good elimination profile due to a greater ability to interact with alveolar immune phagocytes.

## 4 Experimental Section

### Carbon‐Based Materials Preparation and Characterization

Large (LGO) and Small GO (SGO) sheets were produced from graphite powder using the modified Hummers’ method^[^ ^35^ ^]^ and then suspended in sterile water (Gibco, Thermo Fisher Scientific) before characterization (Figure 1). Full characterization of the GOs was performed as previously reported.^[^ ^28^ ^]^

Large (LFLG) and Small FLG (SFLG) sheets were synthesized by exfoliation of graphite (Sigma Aldrich, LOT #BCBS5850V) using riboflavin‐5′‐phosphate as previously described.^[^ ^36^ ^]^ Briefly, graphite was dispersed in a 1 mg.mL^−1^ solution of riboflavin 5‐phosphate sodium salt (Rib) in milli‐Q RNA‐free water (1 g in 400 mL) and sonicated via cup horn sonication (Ultrasonic VC505) for 45 min (20% amplitude, 1 s sonication, and 1 s pause). The obtained dispersion was then centrifuged at 5000 RCF, collecting separately the supernatant and the precipitate. The supernatant was filtered on Millipore filters PTFE (pore size <100 µm), washed with water (200 mL) and ethanol (10 mL), and redispersed in water by short bath sonication to produce the Small FLG dispersion. The precipitate was dispersed again in 1 mg.mL^−1^ solution of Rib, sonicated for 10 min by cup horn sonication and centrifuged at 2000 RCF. In this case, after purification, the supernatant was discarded because of the small lateral size of the flakes. The precipitate was submitted to a new cycle of sonication and centrifugation at 500 RCF, to produce after purification the Large FLG dispersion. The dispersions were carefully stored in sealed glass vials in milli‐Q RNA‐free water until use.

The thickness of the GO and FLG flakes was experimentally determined using AFM (Bruker MultiMode 8) equipped with the NanoScope v1.9 software for analysis (Figure 1a). Size distribution of GOs was evaluated using SEM (Figure 1b) as reported previously.^[^ ^28^ ^]^ The lateral size of FLGs was evaluated by a statistical analysis on at least 200 TEM images, using a Hitachi 7500 transmission electron microscope (Hitachi High Technologies Corporation, Tokyo, Japan) equipped with an AMT Hamamatsu digital camera (Hamamatsu Photonics, Hamamatsu City, Japan) (Figure 1c). Raman spectra of GO and FLG sheets drop casted on quartz slides (Electron Microscopy Sciences, Inc.) were acquired using a Raman confocal system (XploRA Plus, HORIBA), equipped with a 638 nm LASER (slit at 100 and hole at 100 for GO; slit at 300 and hole at 500 for FLG) (Figure 1d). For FLG, the shape of the 2D band was analyzed using a Lorentzian distribution to estimate the numbers of layers.^[^ ^68^ ^]^ Briefly, monolayer graphene was made of a single Lorentzian fit, with a half peak width of ≈20–30 cm^−1^. The 2D peak of multilayer graphene was made of two to more components, causing a clear increase in peak width (Figure S5, Supporting Information). In addition, the 2D/G band ratio was determined for FLGs to confirm the thickness of the flakes; a 2D/G ratio > 1.5 indicates monolayer while a ratio < 1.2 indicates few‐layer. The main physicochemical properties of GOs and FLGs are summarized in the Figure 1e.

Multi‐walled carbon nanotubes (positive control; Mitsui, Japan, type Mitsui‐7; kind gift from Prof. Ulla B. Vogel, National Research Centre for the Working Environment, Denmark) were heated at 180–200 °C for de‐pyrogenization overnight, dispersed in 0.5% bovine serum albumin (BSA) in water for injection (Gibco, Thermo Fisher Scientific), and then sonicated in a water bath for 5–7 min at 80 W (VWR essential, UK).

Endotoxin levels were measured for all materials following the method described in Mukherjee et al.^[^ ^38^ ^]^ based on primary macrophages secretion of TNF‐*α* and showing absence of significant contamination (data not shown).

### In Vivo Exposure by Oropharyngeal Aspiration

C57BL/6J mice (6–8 week old, female; Envigo, UK) were housed in ventilated cages with access to food and water ad libitum in an environment controlled for humidity, temperature, and light. All experiments were performed in compliance with UK Home Office Animals (Scientific Procedures) Act 1986 (ASPA 1986) and under the project license no. P089E2E0A, reviewed by the University of Manchester Animal Welfare and Ethical Review Body. Animals were exposed by single oropharyngeal aspiration to vehicle (water for injection, Gibco, ThermoFisherScientific) or 30 µg of materials suspended in 30 µL of vehicle. Prior to administration, mice were anaesthetized by inhalation of 4% isoflurane and then held on a slanted board. The animals (3–4 animals per group) were kept for 1, 7, and 28 days after the single exposure.

### Bronchoalveolar Lavage Fluids and Lungs Collection

At day 1, 7, and 28 after exposure, mice were euthanized by IP injection of pentobarbitone (100 µL/animal). Left lungs were kept unwashed by ligaturing the respective trachea, and right lungs were washed with ice cold PBS (3 times 800 µL) to collect broncho‐alveolar lavages (BAL) fluids. Left lungs were collected and inflated with pure formalin (40% formaldehyde) for further histopathological analysis.

### Phenotyping of BAL cells

BAL cells were centrifuged at 1500 rpm for 5 min at room temperature (Hettich GmbH) and supernatants were stored at −80 °C. Cells were then suspended in PBS (Merck‐Sigma), counted using a haemocytometer (Marienfeld GmbH), and an equal number per animal and condition was cyto‐spun on a glass slide (Superfrost plus slides Thermo Fisher Scientific) at 600 rpm for 5 min (Hettich GmbH). Cells on slides were fixed in 100% ice‐cold methanol for 10 min, dried and stored at −20 °C. Differential cell staining was performed using Diff‐Quick stain (Epredia Shandon Kwik‐Diff Stains) following the provider's instructions. Neutrophils, eosinophils, mono‐ and multi‐nucleated macrophages, and lymphocytes were identified and counted using bright field optical microscopy (AxioObserver, Zeiss). Values are expressed as percentage of each cell type for 500 cells counted per animal and condition (Figure 2a), or multiplied by the total number of cells present in the BAL fluid (prior to cyto‐spinning) to report the total number of each cell type (Figure 2b).

### Histopathology Analysis

Inflated lungs were fixed in 10% formalin (4% formaldehyde; Merck–Sigma) for 24 h and then transferred in 75% ethanol (Thermo Fisher Scientific) prior embedding in paraffin. Embedded lungs were cut using a microtome (RM2255, Leica Biosystems), and 5.0 µm thick sections were put on glass slides (Superfrost plus slides, Thermo Fisher Scientific). Lung sections were stained with Hematoxylin and Eosin using an automatic stainer (XL autostainer, Leica Biosystems). For the histopathological analysis of the lungs, bright field images were acquired with a slide scanner (Pannoramic 250 Flash, 3DHistech Ltd). Immune cell infiltrates and granuloma‐like structures were identified, and the bronchial thickness was measured. All the histopathological analyses were performed using CaseViewer (software version 2.4.0.11902, 3DHistech Ltd).

### Evaluation of GBM Presence and Degradation Using Raman Spectroscopy

To evaluate the distribution of GBMs in lung sections and BAL cells, Raman spectroscopy‐based confocal microscopy (XploRA Plus, HORIBA) was performed using GBM specific Raman signatures. Prior to scanning, lung sections embedded in paraffin on glass slides (Superfrost plus, Thermo Fisher Scientific) were dewaxed, whereas ice‐cold methanol fixed BAL cells cyto‐spun on glass slides were prepared as mentioned above. In both cases, scanning was performed by Raman microscopy at a wavelength of 638 nm and a grating of 600, with 3 µm of distance between each scanning point.

The parameters used to identify materials were adapted to GBM Raman spectral characteristics (D and G bands for GO; G and 2D bands for FLG) to optimize the detection. A slit and hole of 100 µm were used for GO, whereas a slit of 300 µm and a hole of 500 µm were used for FLG. GBMs were identified based on their Raman signatures: D (≈1335 cm^−1^) and G (≈1590 cm^−1^) bands for GO, as well as 2D (≈2650 cm^−1^) band for FLG. However, only the intensity of the G band was used for overlapping Raman maps and bright‐field images (Figure 4a; Figure S3a,b, Supporting Information). Background removal was performed to remove the noise attributed to the biological signature of BAL cells or lung tissues. Raman signatures obtained before and after background removal are presented in Figure S4 (Supporting Information) and Figure 5, respectively.

After Raman scanning, the same BAL cell slides were stained with Diff‐Quick stain (Kwik‐Diff, Shandon, Thermo Fisher Scientific) in order to overlap the Raman maps (G band only) with the optical microscopy images of the stained immune cells and semi‐quantify the level of internalization by either neutrophils or macrophages. The number of neutrophils and macrophages present on each slide was determined by counting 150 cells per animal for each condition. The number of Raman positive and Raman negative cells for GOs and FLGs was evaluated for both cell types. For each positive cell, the relative quantity of material internalized per cell (i.e., average cell loading) was based on the number of pixels positive for GBM Raman signature and expressed as % of the total number of pixels that represents the total surface of the considered cell (i.e., 100%). To estimate the overall “total material loading” per cell type (either macrophages or neutrophils) and condition (SGO, LGO, SFLG, and LFLG), the percentage of positive cells was multiplied by the average loading in each positive cell in order to take into consideration the negative cells in the analysis. To measure the proportion of macrophages and neutrophils involved in the elimination of GBMs, the total loading values for either macrophages or neutrophils (expressed as % of cell surface) were multiplied by the respective number of cells measured in BALF at day 1 (Figure 4a). To normalize the results per surface area, values obtained for neutrophils were divided by two in order to take into consideration a two‐time smaller total cell surface compared to macrophages. Finally, total values (macrophages and neutrophils combined) were calculated to determine the percentage of internalization in either macrophages or neutrophils in respect to the total amount of material internalized by BALF cells (i.e., the two cell types combined).

Alveolar clearance over time (i.e., material elimination at day 7 and 28 in comparison to day 1, used as starting point) was evaluated using the calculated total material loading in BALF cells at day 1, 7, and 28. For day 1, the total material loading in cells combined the results of both macrophages (at day 1) and neutrophils (at day 1). To set the total loading at 1 day at 100%, the total material loading in macrophages and the total material loading in neutrophils (normalized by the proportion of neutrophil in BAL and by the surface of neutrophils/macrophages ( = 0.5)) for each animal were summed. To have a standard deviation, the value obtained for each animal was divided by the average total loading in macrophages and neutrophils of the 3 animals. For day 7 and 28, total material loading was based on loading of materials in macrophages only (as neutrophils were no longer detected in BALF at day 7 and 28). Values of remaining materials (%) in BALF cells at day 7 and 28 were normalized to the total quantity of materials calculated at day 1 in the combined macrophages and neutrophils (i.e., values at day 1 were set at 100%).

Raman microscopy was also used to evaluate GBM intracellular degradation in BAL cells (macrophages and others cells) over time (from 1 to 28 days after exposure), based on the evolution of the D and G band intensities for all cells present in the ROI of the Raman scan. All the positive spectra identified by Raman spectroscopy were considered for the analysis. To evaluate material degradation, variations in calculated *I*_D_/*I*_G_ ratio and in Raman peak intensities and changes in spectral structure were considered.

### Statistical Analysis

Data were expressed as mean ± standard deviation. Statistical analysis was performed using Graphpad Prism 8.0 (GraphPad Software Inc., San Diego, CA). For BALF analysis and histopathology, two‐way ANOVA followed by Dunnett's post‐hoc test was used to evaluate differences compared to the negative control (*n* = 3–4). As MWCNT exposures were very positive for most endpoints tested, an extra analysis was performed separately to avoid false negative results for other materials. For Raman analysis, one‐way ANOVA (for neutrophils) or two‐way ANOVA (for macrophages) followed by Tukey's multiple comparisons test were used to evaluate at each time‐point statistical differences of GBM internalization between respective large and small sizes or between GO and FLG (*n* = 3).

## Conflict of Interest

The authors declare no conflict of interest.

## Author Contributions

T.L. And L.A.V.de L. contributed equally to this work. T.L. performed conceptualization (lead), investigation (lead for biological analyses), formal analysis (lead), methodology (lead), visualization (lead), writing – original draft (lead), and writing – review and editing (lead). L.A.V.de L. performed investigation (lead for biological analyses), formal analysis (lead), methodology (lead), visualization (lead), and review and editing (equal). M.A.L. performed investigation (lead of FLG materials) and characterization (lead for FLG materials). A.F. performed characterization (lead for GO materials) and investigation (technical assistance). N.L. performed investigation (lead for materials), resources (lead for GO materials), formal analysis (lead for GO materials), visualization (lead for materials), supervision (supporting), and writing – review and editing (equal). A.B. performed conceptualization (lead), resources (lead for FLG materials), funding acquisition (lead), supervision (lead), and writing – review and editing (equal). K.K. performed conceptualization (lead), resources (lead for GO materials), funding acquisition (lead), supervision (lead), and writing – review and editing (equal). C.B. performed conceptualization (lead), investigation (supporting), resources (lead for MWCNT materials), funding acquisition (lead), supervision (lead), visualization (supporting), and writing – review and editing (equal).

## Supporting information

Supporting Information

## Data Availability

The data that support the findings of this study are available from the corresponding author upon reasonable request.
