# Supplementary material for: Lung Persistence, Biodegradation, and Elimination of Graphene‐Based Materials are Predominantly Size‐Dependent and Mediated by Alveolar Phagocytes
Source: Small. 2023 Jun 1;19(39):2301201. doi: 10.1002/smll.202301201 (PMC11475755; doi:10.1002/smll.202301201)
Supplement: Supplementary file 1 — Supporting Information [file SMLL-19-2301201-s001.pdf]

NANO · MICRO
small

Supporting Information

for *Small*, DOI 10.1002/smll.202301201

Lung Persistence, Biodegradation, and Elimination of Graphene-Based Materials are Predominantly Size-Dependent and Mediated by Alveolar Phagocytes

*Thomas Loret, Luis Augusto Visani de Luna, Matteo Andrea Lucherelli, Alexander Fordham, Neus Lozano, Alberto Bianco**, Kostas Kostarelos* and Cyrill Bussy**

Supporting Information

Supplementary Figures

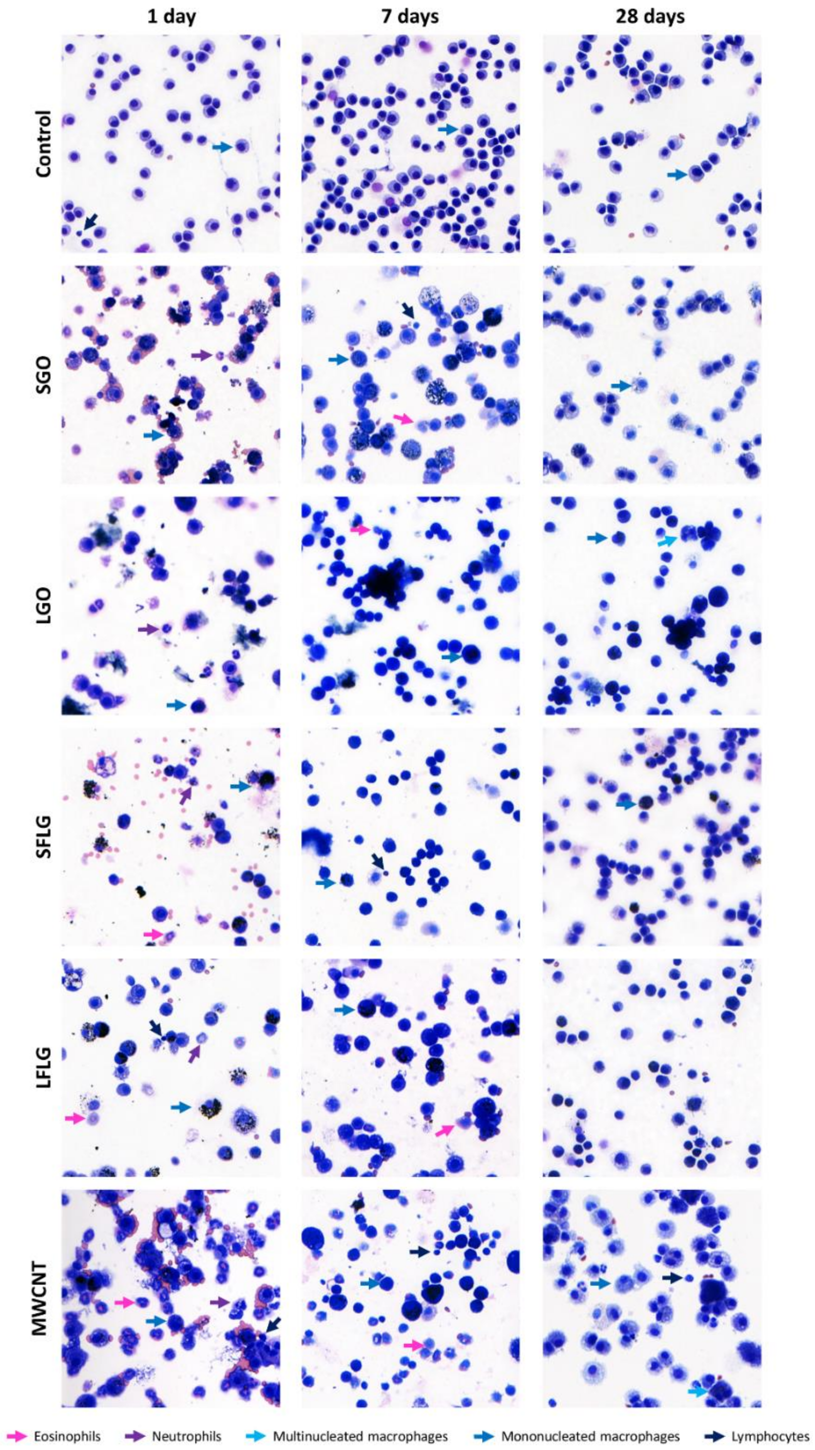

Figure S1. Phenotyping of Bronchoalveolar lavage (BAL) cells

Mice were exposed by oropharyngeal aspiration to 30 µg of SGO, LGO, SFLG, LFLG, MWCNTs or vehicle control. BAL cells were collected at day 1, 7 and 28, counted and cytospun on glass slides. Cells were stained, phenotyped and the percentages of eosinophils (pink arrow), neutrophils (purple arrow)

mono- (cyan arrow) and multi-nucleated macrophages (french Blue), and lymphocytes (midnight blue) were evaluated.

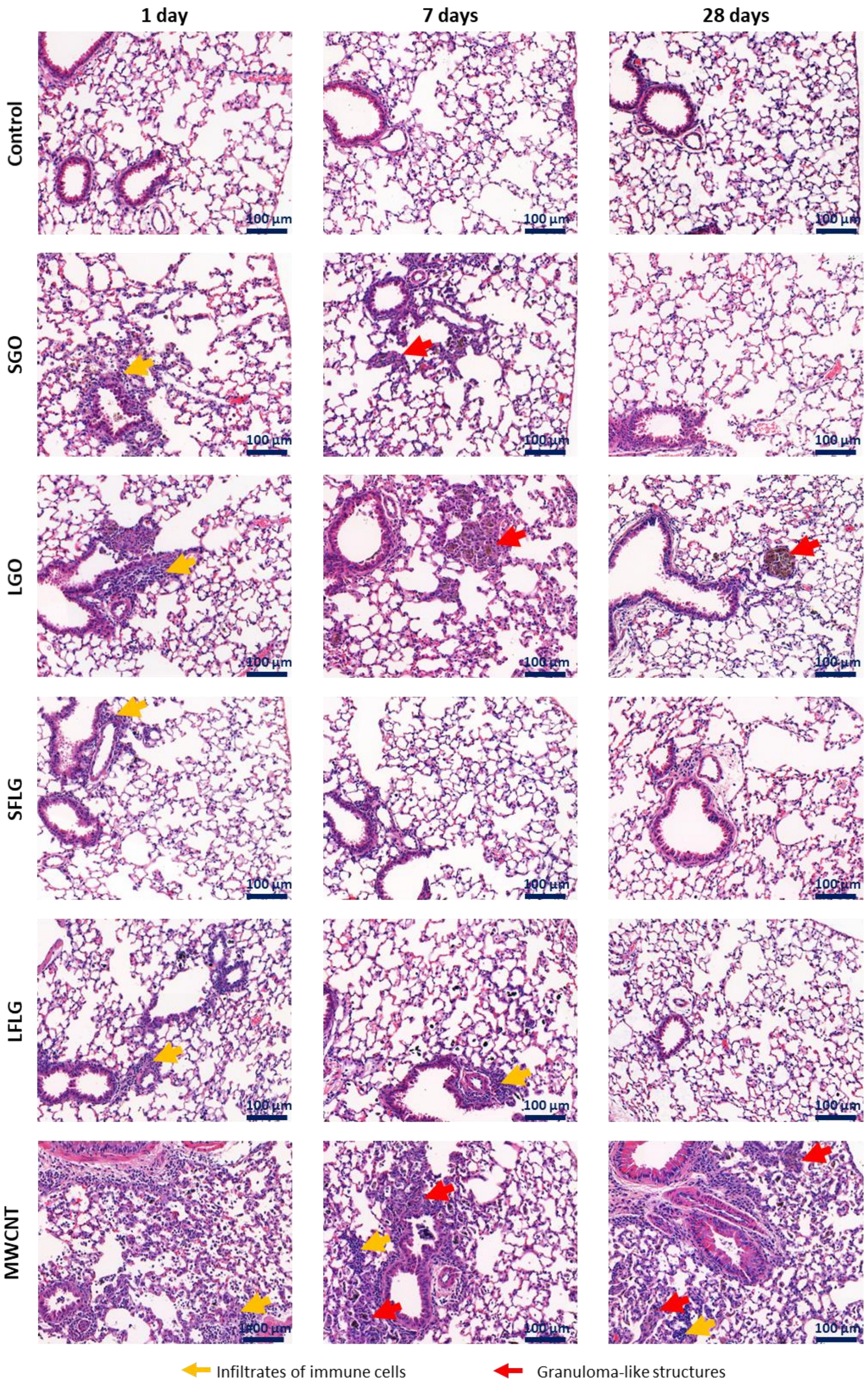

Figure S2. Tissue changes in comparisons to negative and positive controls

Mice were exposed by oropharyngeal aspiration to 30 µg of SGO, LGO, SFLG, LFLG, MWCNTs or vehicle control. Left lungs were collected at day 1, 7 and 28, inflated with formalin, embedded in paraffin and then processed. Lung sections (5 µm) were stained in Haematoxylin-Eosin for

histopathological analysis. Immune infiltrates (red arrow) and granulomatous-like structures (yellow arrow) were identified.

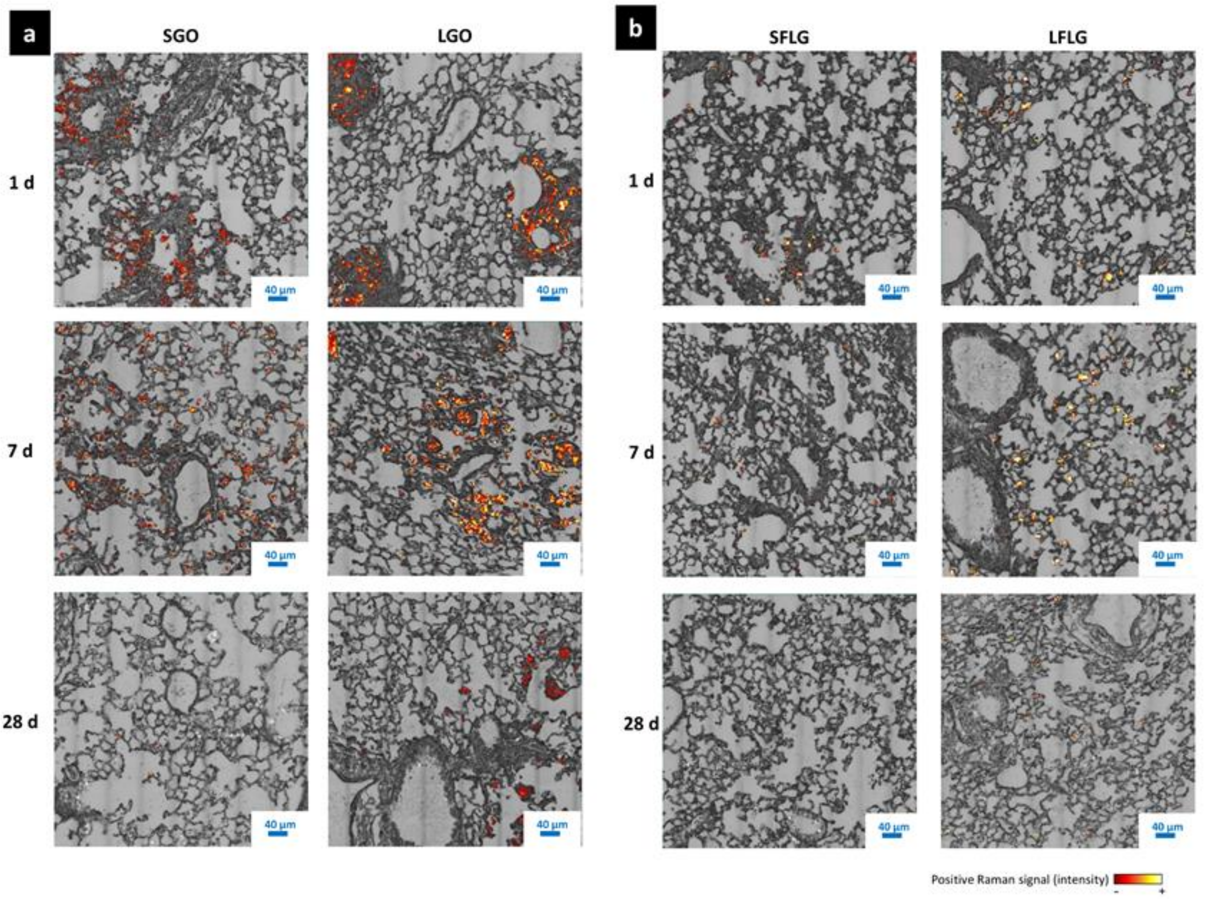

Figure S3. **Evaluation of GBM distribution in lung tissues**

Mice were exposed by single oropharyngeal aspiration to 30 µg of GBMs. One, 7, 28 days after the last exposure, left lungs were collected, inflated with formalin, embedded in paraffin and then processed (n=3). Lung sections (5 µm) were dewaxed and scanned using Raman confocal microscopy. GBMs were identified based on their Raman signatures. Overlay of Raman intensities for the G band and bright field images are presented in the figure: (a) representative Raman lung map for SGO and LGO materials. (b) representative Raman lung map for SFLG and LFLG materials

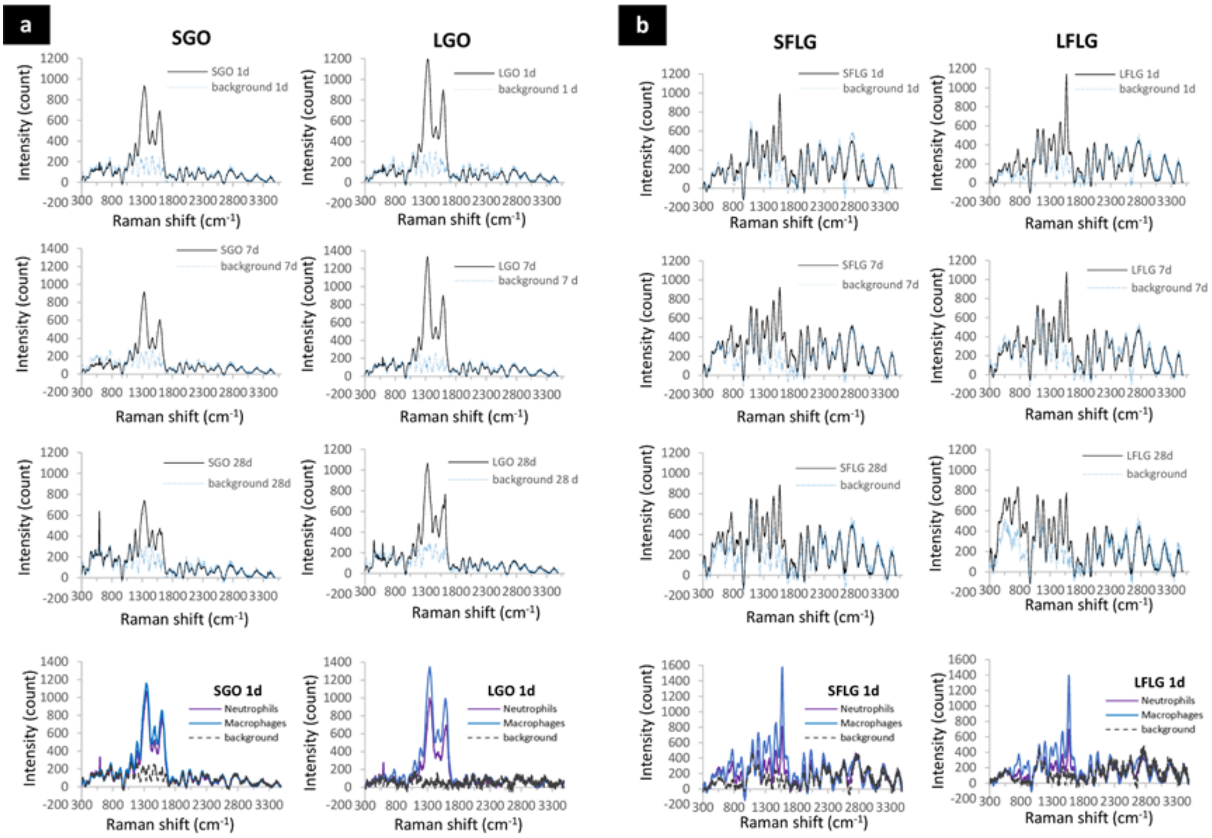

Figure S4. GBM clearance and intracellular degradation over time before background removal

Mice were exposed to 30 µg of GBMs and alveolar cells were collected at day 1, 7 and 28. Alveolar cells were analysed by Raman and stained for cell phenotyping to evaluate the intracellular degradation in alveolar phagocytes. To evaluate GBM degradation, the average intensities of all positive Raman signatures were calculated for each animal. Unprocessed spectra as well as background intensities attributable to the biological signatures of the cells are indicated in the graphs. For each material and time-point, a corresponding average intensities for 3 animals is presented in the graphs. Two hundred to 3000 positive spectra were recorded for each animal, depending on the amount of material detected. (a) representative Raman spectra for SGO and LGO materials. (b) representative Raman spectra for SFLG and LFLG materials

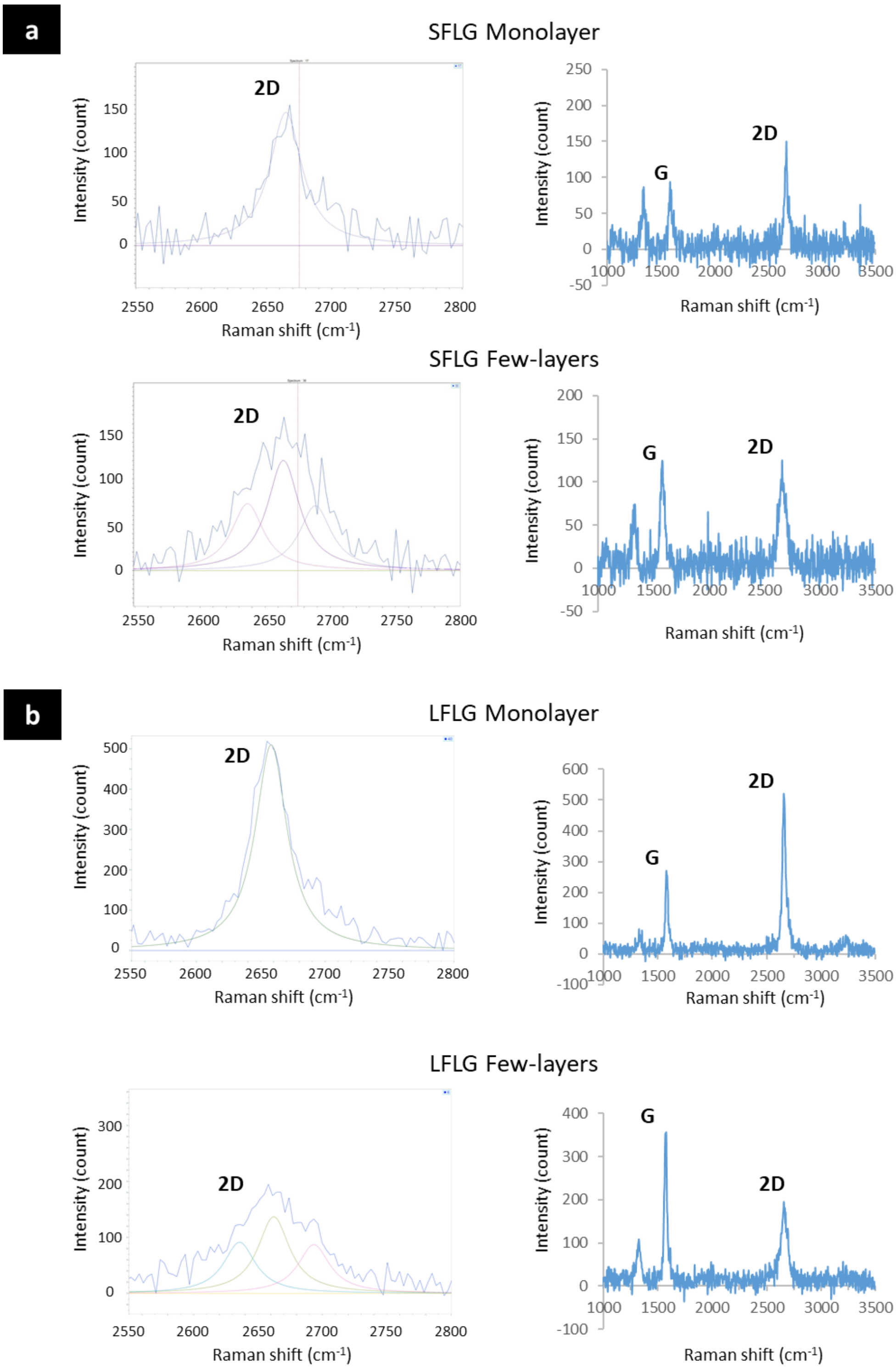

Figure S5. Examples of evaluation of the number of layers of FLGs using Raman

The shape of the 2D peaks of the SFLG (a) and LFLG (b) were analysed using Lorentzian distribution to estimate the numbers of layers (100 sheets were analysed). Monolayers were composed of a single Lorentzian fit, with a half peak width of around 20-30 cm−1. The 2D peaks of few-layer graphene were composed of two to more components, causing a clear increase in peak width. 2D/G ratio of FLGs were evaluated to confirm the monolayer's statute; the 2D/G ratio being > 1.5 for monolayers and < 1.2 for few-layers.
